# Tear Levels of Inflammatory Cytokines in Keratoconus: A Meta-Analysis of Case-Control and Cross-Sectional Studies

**DOI:** 10.1155/2021/6628923

**Published:** 2021-09-30

**Authors:** Huan Zhang, Xiupeng Cao, Ying Liu, Peihong Wang, Xuan Li

**Affiliations:** ^1^Clinical College of Ophthalmology, Tianjin Medical University, Tianjin, China; ^2^The First People's Hospital of Neijiang, Sichuan, China; ^3^Southwest Medical University, Luzhou, China; ^4^Tianjin Eye Hospital, Tianjin Eye Institute, Tianjin Key Laboratory of Ophthalmology and Visual Science, Tianjin, China; ^5^Nankai University Affiliated Eye Hospital, Tianjin, China

## Abstract

**Purpose:**

To assess the tear levels of inflammatory cytokines in patients with keratoconus (KC).

**Design:**

Systemic review and meta-analysis.

**Methods:**

The following electronic databases and search engine were searched: PubMed, EMBASE, Web of Science, and Google Scholar. A systematic search of all relevant studies published through January 2021 was conducted, and the standardized mean difference (SMD) and 95% confidence interval (CI) of cytokine levels were calculated to estimate the pooled effects. Sensitivity analysis, subgroup analysis, and metaregression were applied to explore the sources of heterogeneity.

**Results:**

A total of 7 studies with 374 participants (374 eyes) from clinical studies were included. The tear levels of interleukin-1 beta (IL-1*β*), interleukin-6 (IL-6), and tumor necrosis factor alpha (TNF-*α*) were significantly increased in KC compared with normal controls. The SMD of IL-1*β* was 1.93 (95% CI 0.22 to 3.65, *P* = 0.03). The SMD of IL-6 was 1.22 (95% CI 0.59 to 1.84, *P* < 0.001). The SMD of TNF-*α* was 1.75 (95% CI 0.66 to 2.83, *P* = 0.002). There was no significant difference between the two groups on interleukin-4 (IL-4) and interleukin-10 (IL-10). The SMD for IL-4 was 2.36 (95% CI -0.28 to 5.00, *P* = 0.08) and for IL-10 was 0.30 (95% CI -1.29 to 1.89, *P* = 0.71). Meta-regression analysis indicated that the heterogeneity maybe significantly correlated with the method of detection, the different ages, and the source of population.

**Conclusions:**

Our meta-analysis demonstrated that proinflammatory cytokines IL-1*β*, IL-6, and TNF-*α* were increased, indicating that cytokine profile changed in KC tears and inflammation may play an important role in the pathogenesis and development of KC.

## 1. Introduction

Keratoconus (KC) is a progressive corneal ectasia disease characterized by corneal thinning, conical corneal protrusion, and irregular astigmatism that can lead to serious visual impairment [1]. Currently, KC is regarded as a multifactorial and complex disease involving the interaction of variable genetic and environmental factors [2]. Twins and family studies provide profound evidence of the genetic role in keratoconus development [3, 4]. Eye rubbing, allergy, asthma, and atopic dermatitis are important environmental risk factors for KC [5–8]. KC is the most common cause of corneal transplantation in developing countries, and the prevalence of in the whole population is 1.38 per 1000 population [8]. This disorder usually affects bilateral eyes, resulting in irreversible visual impairment and the quality of life decline in patients. Although several treatments including rigid gas permeable contact lens, sclera lens, intrastromal corneal ring segments, and corneal cross-linking are available for the early to intermediate stages [9, 10], patients have to choose keratoplasty due to corneal scarring and the risk of secondary corneal perforation in some advanced cases. It is essential to elucidate the definite mechanism of keratoconus progression to explore new treatments. Despite numerous studies in the last several decades, the mechanisms of KC development and progression remain unclear.

KC has no obvious inflammatory features, such as neovascularization and inflammatory cell infiltration. It therefore was defined as a noninflammatory degenerative disease in the past [1]. On the contrary, multiple recent studies have shown that chronic inflammation may be a novel direction in the mechanisms of keratoconus progression [11–16]. Furthermore, increasing investigations into disease pathogenesis have implicated the role of oxidative stress-induced inflammation in disease progression. There were two main viewpoints on mechanism of oxidative stress in KC: one was defects in reactive oxygen species (ROS) removal that caused by downregulation of antioxidant enzyme expression in corneal tissue [16, 17], and another was dysfunction of mitochondria and dysregulated autophagy lead ROS to increase [15, 18, 19]. Mitochondria are also the primary source of cellular ROS and are therefore highly involved in oxidative stress [20]. An abundance of evidence points to a role for ROS generated by mitochondria in regulating inflammatory signaling, driving a chronic inflammation [21–24].

Many previous studies have also found that KC is associated with imbalances and abnormalities of inflammatory cytokines (inflammatory mediators) in local microenvironment [25–28]. Tear film is an important part of the ocular surface microenvironment, and its homeostasis directly affects corneal health, since the related studies on corneal tissue only represent advanced cases, as corneal tissues usually derived from patients with advanced cases with keratoplasty. Tear fluid is a more representative biological sample, which can better reflect the inflammatory state in the microenvironment of KC with different stages than corneal tissue. Accumulating evidence supported that cytokine level was abnormal in KC tears. These cytokines mainly include interleukin- (IL-) 1*β*, IL-4, IL-6, IL-10, and tumor necrosis factor- (TNF-) *α*. IL-1*β* is a proinflammatory cytokine, an alarmin which, once released into the extracellular environment, triggers the inflammatory response [29]. IL-6 is a pleiotropic cytokine, and its role as an inflammatory mediator has been proved in many diseases with immunological basis [30, 31]. IL-6 is important for regulating B cell and T cell responses and for coordinating the activity of the innate and the adaptive immune systems [32]. TNF-*α* has been identified as a major regulator of inflammatory response, which is functionally known to trigger a series of various inflammatory molecules, including other cytokines and chemokines [33]. Physiologically, TNF-*α* is a crucial component of normal immune response and can activate the immune system to regulate. However, inappropriate or excessive production of TNF-*α* may be harmful and lead to diseases [34]. IL-1*β*, IL-6, and TNF-*α* have been proved to be involved in the pathogenesis of various chronic inflammation and autoimmune diseases [29, 32, 33, 35, 36]. IL-4 is a pleiotropic cytokine, and it regulates the differentiation of naive CD4+ T cells into helper Th2 cells, which favor a humoral immune response. Another dominant function of IL-4 is the regulation of immunoglobulin class switching to the IgG1 and IgE isotypes. Excessive IL-4 production by Th2 cells has been associated with elevated IgE production and allergy [37]. IL-10 is an important anti-inflammatory cytokine, which is mainly secreted by Th2 cells and promotes the differentiation of macrophages to M2 phenotype, and inhibits the release of proinflammatory mediators, including TNF-*α*, IL-1*β*, IL-6, and IL-8 [38]. Balasubramanian et al. [39] showed that IL-4, IL-6, IL-10, and TNF-*α* all significantly increased in KC tears. Jun et al. [40] reported that the tear level of IL-6 increased, and TNF-*α* and IL-4 decreased in KC compared with healthy controls. Pásztor et al. [41] found a decrease of IL-6 in KC tears. Thus, the potential inflammatory pathway in KC pathological mechanism has been proposed [42, 43]. However, it remains unclear about the expression characteristics of inflammatory factors in KC tear and whether the tear environment is in an inflammatory state. It is essential to investigate the pathological mechanism of keratoconus in order to explore new treatments. Hence, we performed a meta-analysis for the tear levels of these cytokines to investigate the inflammatory state in the tear environment of KC.

## 2. Materials and Methods

This study was registered prospectively in the International Prospective Register of Systematic Reviews (PROSPERO) (CRD42020154426) and followed Preferred Reporting Items for Systematic Reviews and Meta-analyses (PRISMA) reporting guideline, as illustrated in Table 1.

### 2.1. Search Strategy

Three international databases (Web of Science, PubMed, and EMBASE) and Google Scholar were searched for relevant published articles from inception to January 2021. All studies that compared the tear levels of inflammatory mediators between KC and control groups (healthy) were searched. Searches were restricted to English language. The keywords were “keratoconus” and “inflammation” or “inflammatory mediators” or “cytokines” or “proinflammatory cytokines.” In addition, the reference lists of relevant articles were scanned for articles of interest.

### 2.2. Inclusion and Exclusion Criteria

Articles from peer-reviewed medical journals were included if they reported on studies meeting the following criteria: (a) the design of the study was case-control or observational cross-sectional study in human; (b) the case group must be untreated KC patients; (c) the control group must be health control with or without mild or moderate myopia; (d) the outcomes must be the levels of cytokines in tears; (e) the number of participants was more than ten; (f) all participants must stop wearing contact lens at least one week before sampling tears. Studies excluded were as follows: (a) studies with any confounding factors that affected test levels of cytokines including systemic or local active inflammation, systemic or local infectious disease, current treatment with systemic or local anti-inflammatory drugs, history of ocular surgery, systemic or localized allergy, autoimmune disease, and dry eye; (b) vitro or animal experiments; and (c) case-report, review, meta-analysis, comments, or conference papers.

### 2.3. Data Extraction

The data was independently extracted by two investigators using a predefined data extraction form. Extracted information includes the following: title, study author, year of publication, country, gender, number of eyes, stage of KC, design of study, detection method, outcome (tear levels of cytokines), and contact lens worn.

### 2.4. Quality Assessment

The methodological quality of the included studies was evaluated using Newcastle-Ottawa Quality Assessment Scale (NOS) and 11-item checklist which was recommended by Agency for Healthcare Research and Quality (AHRQ). For case-control studies, we recommend the use of NOS, and the ARHQ methodology checklist was applicable for cross-sectional studies [44]. Article quality was assessed as follows: for case-control studies, studies with more than six stars are considered high quality; for cross-sectional studies, a score of 0-3 is low quality, 4-7 is moderate quality, and 8-11 is high quality.

### 2.5. Statistical Analysis

Concentration of cytokines is a continuous variable. When similar outcomes were measured with different methods, we calculated the standardized mean difference (SMD) to estimate the effects. The statistic formulas of Luo et al. [45] and Wan et al. [46] were applied to calculate the mean and standard deviation when some studies reported results using the median with first and third quartiles. *Q*-statistic (*P* < 0.05) and *I*^2^ tests (*I*^2^ > 50%) were applied to determine heterogeneity. A value of 25% corresponds to low, 50% to moderate, and 75% to high heterogeneity [47]. The Mantel-Haenszel method for fixed effects and the Der Simonian and Laird method for random effects were used to estimate pooled effects [48]. The random effects model was used to pool the data when the heterogeneity was moderate or high. Data were shown as SMD and 95% confidence interval (CI). Sensitivity analysis was performed to verify the effect of studies on the stability of the summary estimates by excluding each single study. We used a random-effects model for a priori subgroup analysis according to the method for detecting cytokine concentration, Enzyme-linked Immunosorbent Assay (ELISA) vs. non-ELISA (Light Initiated Chemiluminescent Assay (LICA), cytometric bead array (CBA), and cytokine antibody array). Metaregression was performed to explain the between-trial heterogeneity observed (*I*^2^ statistics > 50%). Detection method, unit of measurements (Pg/ml vs. FIU/mg), quality score of study (moderate vs. high quality), region (America vs. Europe vs. Asia vs. Australia), and age (young vs. middle age) were used for metaregression analyses. We had planned to assess publication bias by using funnel plots and Egger's test but were unable to do because of insufficient number of included studies (Cochrane handbook 10.4.3). Review Manager (version 5.3; Cochrane Collaboration, Oxford, United Kingdom) and Stata software (version 15.1; Stata Corp) were used to perform statistical analysis, and *P* < 0.05 was considered as statistically significant.

## 3. Results

### 3.1. Search Results

**Figure 1:**
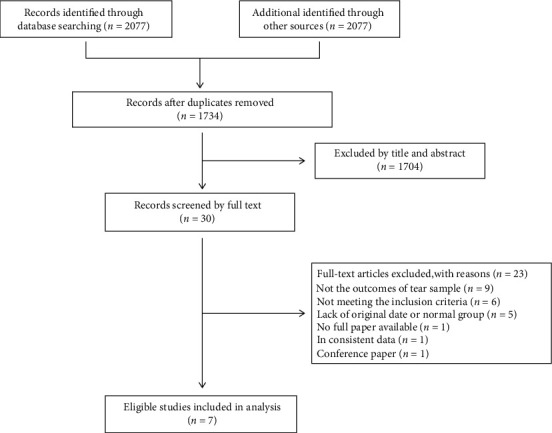
Flow of studies through the meta-analysis.

Our search yielded 2079 articles. Having excluded 345 duplicate records, we screened the remaining 1734 on the basis of title and abstracts and discarded 1704 as irrelevant. For one record of conference papers, only abstracts were available. We contacted the author of the conference abstracts for further information and followed up two weeks later. However, we excluded the study because of no response. A total of 30 full texts were reviewed, of which 7 published studies satisfied the eligibility criteria and were finally included (Figure 1).

### 3.2. Study Characteristics

A total of 7 articles, 374 eyes of 374 subjects (225 for KC and 149 for normal eyes), were included in the study. We included three observational cross-sectional (Lonescu, 2018; Pásztor, 2016; Balasubramanian 2012) and four case-control studies (Sorkhabi, 2015; Lema2009; Lema, 2008; Lema 2005). Of the 7 studies, three were conducted in Spain, and one in the USA, Australia, Romania, and Iran. These six studies reported the gender distribution between KC and controls (Lonescu, 2018; Sorkhabi 2015; Balasubramanian, 2012; Lema2009; Lema, 2008; Lema2005). There was a roughly equal gender distribution between KC and controls in one study only (Lema 2005). Five studies had a higher proportion of male participants in the KC compared to the control group: Lonescu 2018 (64.71% versus 40%), Sorkhabi et al. [29] (57.14% versus 43.33%), Balasubramanian et al. [40] (64% versus 40%), Lema 2009 (70% versus 47.8%), and Lema 2008 (53.6% versus 25%). Three studies reported the proportion of KC in different stages (Lonescu, 2018; Sorkhabi 2015; Lema 2005). The methods measured cytokines were not fully consistent, including ELISA, LICA, cytokine antibody array, and CAB. Different researchers tend to use different detection methods to measure the same outcome. Four studies measured the outcome using ELISA, one by LICA, cytokine antibody array, and CBA. The number of studies including the same outcome was low, ranging from three to seven studies. Participants in four studies did not wear contact lenses (Lonescu, 2018; Sorkhabi, 2015; Lema 2008; Lema2005), and in other three studies, they were asked to discontinue contact lens for 1-3 weeks prior to the sampling. Based on the quality assessment of NOS and the ARHQ methodology checklist, 5 studies were of high quality, while the other two studies were in moderate quality. The defined information was exacted and is summarized in Tables 2–4.

### 3.3. Quantitative Data Synthesis

#### 3.3.1. IL-1*β*

**Figure 2:**
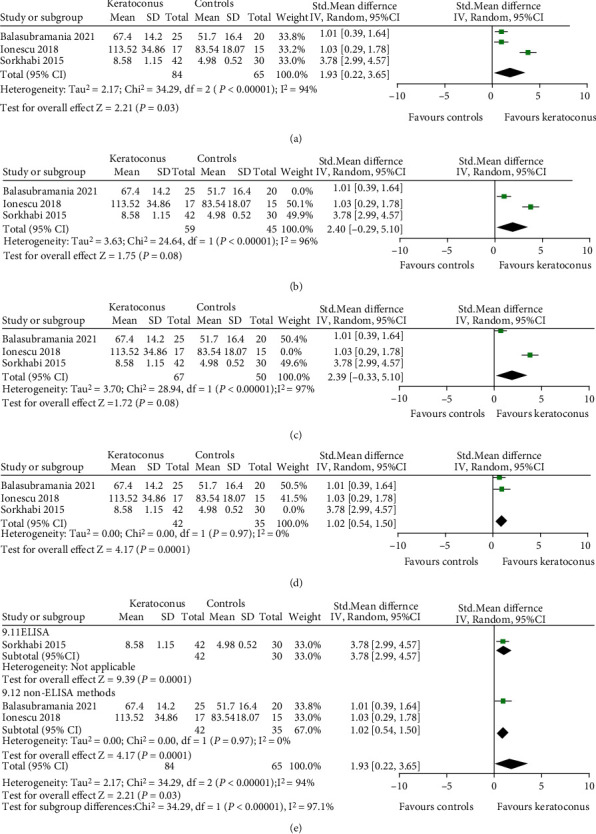
Forest plot for SMD and 95% CI of IL-1*β* in tears by keratoconus versus the control group. (a) The pooled effect of IL-1*β* in all studies. (b–d) Sensitivity analysis of IL-1*β* in tears by omitting one study in each turn. (e) Subgroup analysis of IL-1*β* in tears by detection methods.

Three studies were included which provided quantitative data of IL-1*β* and used different testing methods: LICA (lonescu 2018), cytokine antibody array (Balasubramanian 2012), and ELISA (Sorkhabi 2015). We therefore calculated the effect estimate as the SMD. The meta-analysis findings (Figure 2(a)) showed an increase of IL-1*β* in tears of KC compared with healthy controls (SMD 1.93, 95% CI 0.22 to 3.65, and *P* = 0.03; *I*^2^ = 94%; 3 studies, 149 participants). Sensitivity analysis was performed by excluding each single study. When the study by Balasubramanian et al. [40] or Lonescu 2018 was excluded, the pooled SMD and 95% CI were changed (SMD 2.40, 95% CI -0.29 to 5.10, and *P* = 0.08 or SMD 2.39, 95% CI -0.33 to 5.10, and *P* = 0.08), indicating that these two studies had a great impact on the overall results (Figures 2(b) and 2(c)). When one original study by Sorkhabi et al. [29] was omitted, the heterogeneity was absent (*I*^2^ = 0% and *P* = 0.97) (Figure 2(d)).

We performed subgroup analysis by detection method. The results were consistent with the overall effects. The tear level of IL-1*β* was elevated in both ELISA (SMD 3.78, 95% CI 2.99 to 4.57, and *P* < 0.001) and non-ELISA (SMD 1.02, 95% CI 0.54 to 1.50, and *P* < 0.001) for KC patients (Figure 2(e)).

Metaregression is shown in Table 5. Our results found that region and unit of measurement did not affect the overall effects (both *P* > 0.05), while detection method and quality score of study may be the influencing factors for IL-1*β* concentration in tears (both *P* < 0.001).

#### 3.3.2. IL-4

**Figure 3:**
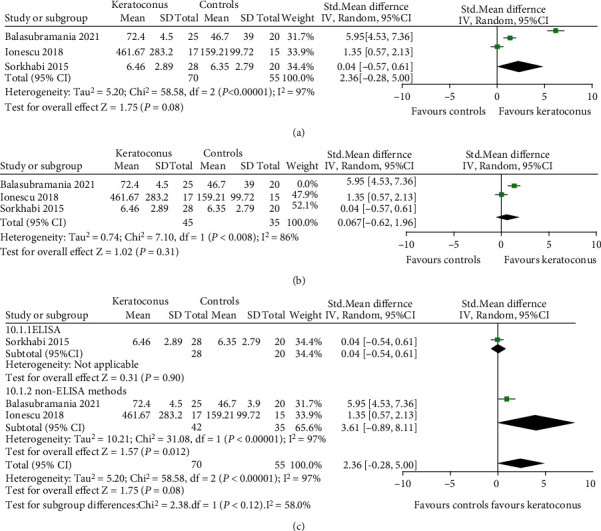
Forest plot for SMD and 95% CI of IL-4 in tears by keratoconus versus the control group. (a) The pooled effect of IL-4 in all studies. (b) Sensitivity analysis of IL-4 in tears by omitting one study in each turn. (c) Subgroup analysis of IL-4 in tears by detection methods.

Three studies provided data on the differential level of IL-4 in tear films between KC and normal. Three studies used different methods: LICA (lonescu 2018), cytokine antibody array (Balasubramanian 2012), and ELISA (Lema 2005). We therefore calculated the effect estimate as the SMD. We converted the reported quartiles for outcome in one study (Lema 2005) into standard deviations. Our results (Figure 3(a)) suggested that there was no significant difference in IL-4 tear level between KC and control groups (SMD 2.36, 95% CI -0.28 to 5.00, and *P* = 0.08; *I*^2^ = 97%; 3 studies, 125 participants). Sensitivity analysis by sequential removing each study, the overall conclusion of the evidence did not change, suggesting that no individual study substantially influenced the pooled effect. When the study (Balasubramanian 2012) was removed, the heterogeneity was partially decreased (*I*^2^ = 86% and *P* = 0.008) (Figure 3(b)).

Subgroup analysis by detection method was performed, and the results were similar to the overall effects. The tear level of IL-4 was not changed in both ELISA (SMD 0.04, 95% CI -0.54 to 0.61, and *P* = 0.90) and non-ELISA (SMD 3.61, 95% CI -0.89 to 8.11, and *P* = 0.12) (Figure 3(c)).

Metaregression is shown in Table 5. Detection method and quality score of study did not affect the pooled effects (both *P* > 0.05), but region and unit of measurement may be influencing factors for IL-4 in tears (both *P* < 0.001).

#### 3.3.3. IL-6

**Figure 4:**
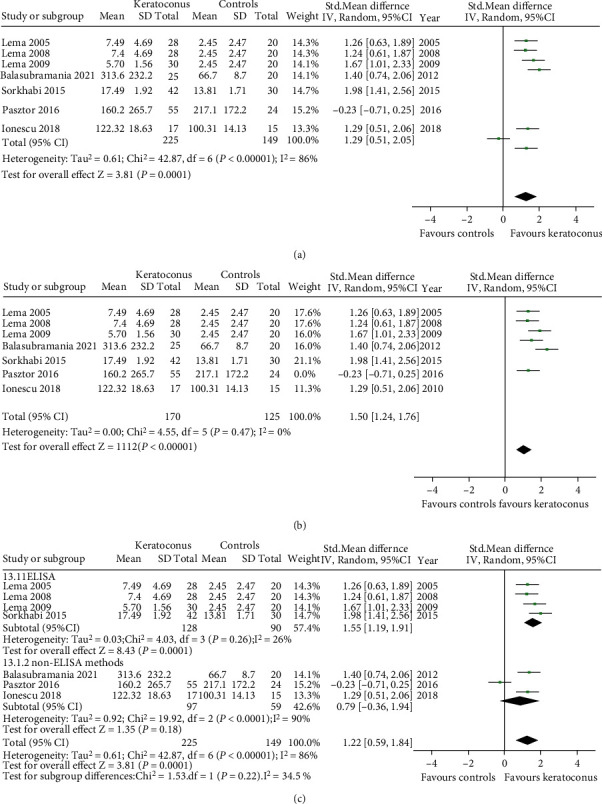
Forest plot for SMD and 95% CI of IL-6 in tears by keratoconus versus the control group. (a) The pooled effect of IL-6 in all studies. (b) Sensitivity analysis of IL-6 in tears by omitting one study in each turn. (c) Subgroup analysis of IL-6 in tears by detection methods.

Seven studies measured IL-6 using different methods: LICA (lonescu 2018), CBA (Pásztor 2016), cytokine antibody array (Balasubramanian 2012), and ELISA (Sorkhabi 2015, Lema 2005, Lema 2008, Lema 2009). We therefore reported the effect size as the SMD of the differential level of IL-6 in tears between KC and controls. We converted the reported median and quartiles for outcome in three studies (Lema 2005, Lema 2008, Lema 2009) into mean and standard deviation. Our results (Figure 4(a)) showed an increase of IL-6 in tears of KC compared with healthy controls (SMD 1.22, 95% CI 0.59 to 1.84, and *P* < 0.001; *I*^2^ = 86%; 7 studies, 374 subjects). Sensitivity analysis by sequential omission of the individual studies did not significantly alter the overall conclusion, suggesting that no individual study substantially influenced the pooled effect. When the study (Pásztor 2016) was excluded, the heterogeneity was absent (*I*^2^ = 0% and *P* = 0.47) (Figure 4(b)).

Subgroup analysis was performed according to the detection method. Stratified for detection method, the tear level of IL-6 was increased in ELISA (SMD 1.55, 95% CI 1.19 to 1.91, and *P* < 0.001), while not significantly changed in non-ELISA (SMD 0.79, 95% CI -0.36 to1.94, and *P* = 0.18) (Figure 4(c)). The factors that caused the changes in statistic results were carefully analyzed. In non-ELISA subgroup, the number of specimens tested in each study was small. Moreover, each study utilized a different detection method. These may result in the great differences in outcomes among studies in non-ELISA subgroup. In the future, it is necessary to carry out more research with a unified test plan.

Metaregression is shown in Table 5. Detection method, unit of measurement, quality score, and region did not affect the overall effects (all *P* > 0.05), but the age may be an influencing factor for IL-6 tear level of KC patients (*P* < 0.001).

#### 3.3.4. IL-10

**Figure 5:**
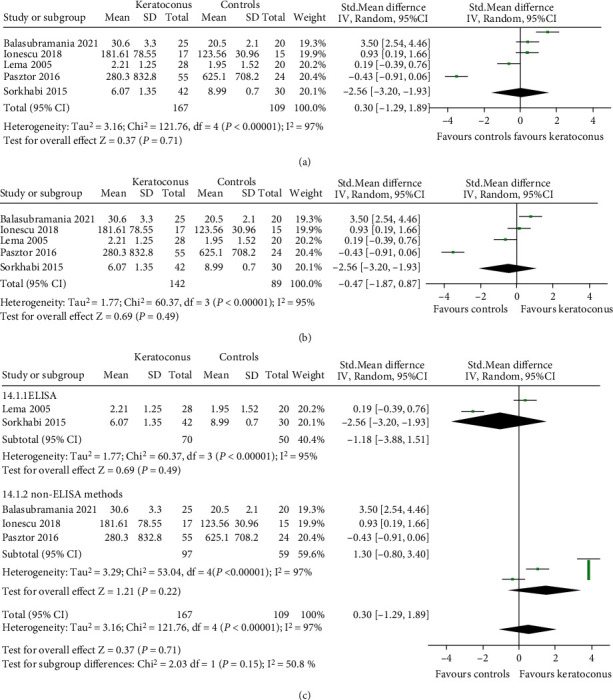
Forest plot for SMD and 95% CI of IL-10 in tears by keratoconus versus the control group. (a) The pooled effect of IL-10 in all studies. (b) Sensitivity analysis of IL-10 in tears by omitting one study in each turn. (c) Subgroup analysis of IL-10 in tears by detection methods.

We included five studies which measured IL-10 by different methods: LICA (lonescu 2018), CBA (Pásztor 2016), cytokine antibody array (Balasubramanian 2012), and ELISA (Sorkhabi 2015, Lema 2005). We therefore reported the effect estimate as the SMD. One study (Lema 2005) provided the median and quartiles which we converted into mean and standard deviation for entering these in the meta-analysis. Our results (Figure 5(a)) showed that the mean tear level of IL-10 was not significantly changed in KC compared with healthy controls (SMD 0.30, 95% CI -1.29 to 1.89, and *P* = 0.71; *I*^2^ = 97%; 5 studies, 276 participants). Sensitivity analysis by individually excluding each study found that the results remained consistent. When the study by Balasubramanian et al. was excluded, it showed that the tear level of IL-10 was slightly lower in the KC group without statistical significance (SMD -0.47, 95% CI -1.81 to 0.87, and *P* = 0.49; *I*^2^ = 95%) (Figure 5(b)).

Subgroup analysis by different detection method was performed. The results were consistent with the overall effects. The IL-10 level was not changed in both ELISA (SMD -1.18, 95% CI -3.88 to 1.51, and *P* = 0.39) and non-ELISA (SMD 1.30, 95% CI -0.80 to 3.40, and *P* = 0.22) (Figure 5(c)).

Metaregression is shown in Table 5. Detection method, region, and age did not affect the overall effects (all *P* > 0.05). The influencing factors for IL-10 were involved in the unit of measurement (*P* = 0.02) and the quality score of study (*P* = 0.03).

#### 3.3.5. TNF-*α*

**Figure 6:**
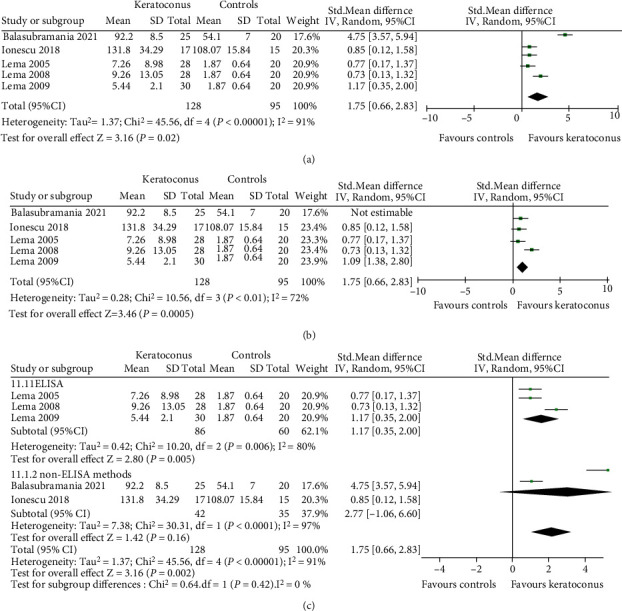
Forest plot for SMD and 95% CI of TNF-*α* in tears by keratoconus versus the control group. (a) The pooled effect of TNF-*α* in all studies. (b) Sensitivity analysis of TNF-*α* in tears by omitting one study in each turn. (c) Subgroup analysis of TNF-*α* in tears by detection methods.

The level of TNF-*α* in the tear film between KC and normal controls was assessed in five studies using different tests: LICA (lonescu 2018), cytokine antibody array (Balasubramanian 2012) and ELISA (Lema 2005, Lema 2008, Lema 2009). We calculated SMD due to the difference in the tests used. Three studies (Lema 2005, Lema 2008, Lema 2009) provided the median and quartiles, which we converted into mean and standard deviation for entering these in the meta-analysis. Our results (Figure 6(a)) showed the proinflammatory cytokine TNF-*α* was higher expression in KC tears than healthy controls (SMD 1.75, 95% CI 0.66 to 2.83, and *P* = 0.002; *I*^2^ = 91%; 5 studies, 223 participants). We performed sensitivity analysis and found that the overall result remained unchanged indicating that no individual study substantially influenced the pooled effect. When the study (Balasubramanian 2012) was excluded, the heterogeneity was decreased (*I*^2^ = 72% and *P* = 0.01) (Figure 6(b)).

Subgroup analysis was performed according to the detection method. Stratified for test method, the tear level of TNF-*α* was significantly increased in ELISA (SMD 1.17, 95% CI 0.35 to 2.00, and *P* = 0.005). However, there was no statistical significance in non-ELISA (SMD 2.77, 95% CI -1.06 to 6.60, and *P* = 0.16) (Figure 6(c)). Factors which influence this statistic outcome were carefully analyzed. There were great differences in outcomes between the two studies in non-ELISA subgroup. One of the main reasons was that each study used a different detection method. Therefore, we need more studies with unified test method to evaluate the tear level of TNF-*α* in KC patients.

Metaregression is shown in Table 5. Detection method and quality score of study did not affect the overall effects (both *P* > 0.05), but the unit of measurement and region might be influencing factors for TNF-*α* (both *P* < 0.001).

## 4. Discussion

The present meta-analysis discussed the levels of five inflammatory cytokines in KC tears, including IL-1*β*, IL-4, IL-6, IL-10, and TNF-*α*. Our results showed that the tear levels of IL-1*β*, IL-6, and TNF-*α* in the patients with KC were significantly higher than those of normal people, while IL-4 and IL-10 did not change significantly, indicating that the tear microenvironment of KC was in an inflammatory state.

For decades, we have never stopped investigating the mechanisms of KC development and progression. The cornea is the outermost avascular and transparent part of the eye consisting of epithelium, Bowman's layer, stroma, Descemet's membrane, and endothelium. Histopathological changes were observed in all layers of KC cornea except for corneal endothelium, mainly including hypertrophy and necrosis of corneal epithelial cells with irregular arrangement, Bowman's layer breaks, the appearance of nonkeratocyte, reduction of keratocyte density, and decrease in the number of lamellae of stroma [49]. Currently, oxidative stress, inflammation, and extracellular matrix (ECM) degradation are considered as the main pathological molecular mechanisms of KC progression [49].

Chronic and prolonged ROS production is considered to be central to the progression of inflammatory diseases [50]. Numerous molecular and biochemical studies have reported imbalance between oxidants and antioxidants in tears, corneal tissues, and cultured keratocytes of KC [14, 51–53]. Atilano et al. [16] found that oxidative stress imbalances were caused by downregulation of the antioxidant enzymes. Excessive oxidants can lead to multiple outcomes such as cell apoptosis, collagen degradation, and activation of proinflammatory cytokines [21, 54, 55]. Multiple reports showed that the cellular source of ROS generated by mitochondria impacted the production of certain inflammatory cytokines [24, 56, 57]; therefore, it is not surprise that mitochondria have been implicated in inflammatory response [23]. Zitvogel et al. [58] found that mitochondria can be considered the principal drivers of NOD-, LRR-, and pyrin domain-containing 3- (NLRP3-) mediated inflammation as they can directly activate the inflammasome complex and represent a checkpoint of the intracellular cascades of numerous downstream pattern recognition receptors (PRRs) [22]; mitochondrial DNA (mtDNA) has been implicated in NLRP3 inflammasome activation, inducing the release of proinflammatory cytokines so strongly [59]. Therefore, KC may be caused by potentially mitochondria dysfunction in a first place, which will eventually increase the release of various downstream proinflammatory cytokines and lead to corneal damage. This need further experiments to investigation. However, whether the expression of various inflammatory cytokines in KC is abnormal is the focus of our review.

Our analysis showed that the tear level of IL-1*β*, IL-6, and TNF-*α* was increased in KC. These cytokines are important as triggers for inflammation and apoptosis. Multiple pathological studies have found apoptosis of corneal epithelial cells and stromal fibroblasts in KC [52, 54]. The proinflammatory cytokines IL-1*β* and TNF-*α* in the local microenvironment have been shown to promote the maturation of Langerhans cells in tissue [60, 61]. Mandathara et al. [62] reported matured Langerhans cells with a significant number in the central cornea in KC suggesting the possibility of active inflammation in KC. Moreover, IL-1*β*, IL-6, and TNF-*α* can upregulate the expression of matrix metalloproteinases (MMPs) in corneal epithelial cells and keratocyte [63, 64]. TNF-*α* also inhibits tissue inhibitor of metalloproteinase-1 (TIMP-1) and TIMP-2 in KC fibroblasts. The elevated cytokines may disrupt the natural balance between proteinases and proteinase inhibitors in favor of the former, engendering pathological degradation of collagen and proteoglycans within the corneal stromal ECM and contributing to the stromal thinning and loss of Bowman's layer which are characteristics of KC. As we all know, IL-4 plays a central role in atopy. Multiple studies have reported that atopic diseases were important risk factors for KC [8, 65]. But our results showed that the tear levels of IL-4 did not change significantly. The reason for this discrepancy may be that our exclusion criteria included studies with atopic or allergic participants. IL-10 is a primarily anti-inflammatory cytokine. Hos et al. [66] showed that TNF-*α* and IL-1*β* were significantly increased, and more severe and prolonged corneal inflammation presented in keratitis animal model of IL-10 deficient. This disruption of the balance between proinflammatory and anti-inflammatory factors may be an important cause of KC progression. Taken together, these findings suggest that IL-1*β*, IL-6, and TNF-*α* play an important role in the pathological mechanism of KC progression.

Moreover, we also performed sensitivity analysis, subgroup analysis, and metaregression for results of the five cytokines to identify potential confounding factors. Sensitivity analysis was also to assess robustness of pooled effects. Sensitivity analysis for IL-4, IL-6, IL-10, and TNF-*α* showed that the overall results were not affected by individual study, suggesting these results have a higher degree of certainty. However, the result of IL-1*β* was not stable enough (Figures 2(b) and 2(c)), and we should treat the conclusions with caution. We performed priori subgroup analysis to identify certain potential influencing factors. Most results of subgroup analysis were consistent with the overall effects. However, subgroup analysis of TNF-*α* and IL-6 by non-ELISA method showed no change in tears of KC compared with healthy controls. We carefully analyzed the reasons for this influence. There were two or three different test methods, and the number of studies was small in non-ELISA subgroup, which could cause these inconsistent results. Therefore, we need more studies with unified test method to evaluate the tear level of TNF-*α* and IL-6 in KC patients. Most results had high heterogeneity. We therefore performed metaregression to explain the between-study heterogeneity observed, and the metaregression model could explain most of these (Table 5). Taken together, test method, unit of measurement, region, and age could be main source of high heterogeneity in inflammatory cytokine tear level of KC patient.

A systematic literature review in 2015 narratively synthesized the evidence of possible inflammatory mediators in cornea, tears, and aqueous humor, suggesting underlying inflammatory pathways in the pathogenesis of KC [42]. Five original studies on tears were included in the review, of which we included three in this meta-analysis. We did not include the remaining studies (Jun 2011 and Pannebaker 2010), because there were confounding factors (atopy and contact lens) to interfere with outcomes and so these two studies were not eligible for inclusion. The authors reviewed four cytokines (IL-1, IL-6, IL-17, and TNF-*α*) in tears. We did not perform the analysis for IL-17 because of the limit number of included studies. This review concluded that the tear level of four cytokines increased in KC, and the imbalance of cytokines in tears would affect tear fluid proteome stability and quality. This finding is consistent with our results. Moreover, this systematic review argued that tear film cytokine alterations do not necessarily reflect intracorneal processes, because the expression of several important mediators has been related to contact lens wear and eye rubbing in KC patients. However, our inclusion and exclusion criteria excluded these confounding factors, such as contact lens, infection, and inflammation.

This meta-analysis showed that the proinflammatory factors IL-1*β*, IL-6, and TNF-*α* in tears of KC were higher than healthy controls, while IL-4 and anti-inflammatory factor IL-10 were not significantly changed, indicating the complex imbalance of proinflammatory and anti-inflammatory factors and inflammatory changes in tears of KC. Although an undetermined causal relationship between the inflammatory state in tears and KC development, we could be certain that altered cytokine profiles were present in KC progression. In addition, Shetty et al. [67] have reported that cyclosporine A can significantly reduce the tear level of MMP-9 and cytokines (IL-6 and TNF-*α*) in tears with concomitant arrest of disease progression in KC patients. Local immunosuppressive treatment on the ocular surface supported the hypothesis that KC progression may involve chronic inflammation and suggested a novel direction of KC treatment. Previous studies have shown that anticytokine antibodies for immune disease treatment achieved good results. Therefore, we should further clarify the relationship between inflammatory cytokines and KC to find novel treatments to arrest the progression of KC in future.

Furthermore, some limitations should be considered. First, the sample size of the original study is limited, so our results may be underpowered and larger sample size should be expected in different populations. Second, insufficient numbers of included studies result in some deficiency: partial subgroup analysis and evaluation of publication bias could not be performed. Third, significant heterogeneity was encountered may due to various test method, valuation technology, age and race of populations enrolled, number of studies, design of study, etc. Although the results of subgroup analysis and metaregression found some of them, we should interpret the results with caution. Fourth, most studies have included KC patients with different stage but have not provided the data of different disease stage, and thus, the inferences were limited again. Finally, teenagers, probably due to their technology gap, were underrepresented in our meta-analysis.

In conclusion, the present meta-analyses demonstrated altered cytokine profiles in tears of KC patients and inflammatory changes on the ocular surface microenvironment. Inflammation may play a crucial role in KC pathogenesis. Further prospective studies with larger sample size, different populations (age, regions, and ethnicity), or different risk factors (genes, eye rubbing, contact lens use, and atopy) are required to elucidate the role of inflammatory cytokines in KC and the relationship between inflammation and KC.

## Figures and Tables

**Table 1:** PRISMA guideline checklist.

Section/topic	#	Checklist item	Reported on page #
Title			
Title	1	Identify the report as a systematic review, meta-analysis, or both.	1
Abstract			
Structure summary	2	Provide a structured summary including, as applicable, background; objectives; data sources; study eligibility criteria, participants, and interventions; study appraisal and synthesis methods; results; limitations; conclusions and implications of key findings; funding for the systematic review; and systematic review registration number.	2
Introduction			
Rationale	3	Describe the rationale for the review in the context of what is already known.	3-5
Objectives	4	Provide an explicit statement of questions being addressed with reference to participants, interventions, comparisons, outcomes, and study design (PICOS).	-
Methods			
Protocol and registration	5	Indicate if a review protocol exists, if and where it can be accessed (such as a web address), and, if available, provide registration information including the registration number.	5
Eligibility criteria	6	Specify study characteristics (such as PICOS, length of follow-up) and report characteristics (such as years considered, language, and publication status) used as criteria for eligibility, giving rationale.	5
Information sources	7	Describe all information sources in the search (such as databases with dates of coverage and contact with study authors to identify additional studies) and date last searched.	2, 5
Search	8	Present the full electronic search strategy for at least one major database, including any limits used, such that it could be repeated.	5
Study selection	9	State the process for selecting studies (that is, for screening, for determining eligibility, for inclusion in the systematic review, and, if applicable, for inclusion in the meta-analysis).	5-6
Data collection process	10	Describe the method of data extraction from reports (such as piloted forms, independently by two reviewers) and any processes for obtaining and confirming data from investigators.	6
Data items	11	List and define all variables for which data were sought (such as PICOS, funding sources) and any assumptions and simplifications made.	6
Risk of bias in individual studies	12	Describe methods used for assessing risk of bias in individual studies (including specification of whether this was done at the study or outcome level, or both), and how this information is to be used in any data synthesis.	-
Summary measures	13	State the principal summary measures (such as risk ratio, difference in means).	6-7
Planned methods of analysis	14	Describe the methods of handling data and combining results of studies, if done, including measures of consistency (such as *I*^2^) for each meta-analysis.	6-7
Risk of bias across studies	15	Specify any assessment of risk of bias that may affect the cumulative evidence (such as publication bias, selective reporting within studies).	-
Additional analyses	16	Describe methods of additional analyses (such as sensitivity or subgroup analyses, metaregression), if done, indicating which were prespecified.	6-7
Results			
Study selection	17	Give numbers of studies screened, assessed for eligibility, and included in the review, with reasons for exclusions at each stage, ideally with a flow diagram.	7, 25
Study characteristics	18	For each study, present characteristics for which data were extracted (such as study size, PICOS, and follow-up period) and provide the citation.	7-8, 22
Risk of bias within studies	19	Present data on risk of bias of each study and, if available, any outcome-level assessment (see item 12).	-
Results of individual studies	20	For all outcomes considered (benefits and harms), present, for each study, simple summary data for each intervention group and effect estimates and confidence intervals, ideally with a forest plot.	8-12, 25-30
Syntheses of results	21	Present the main results of the review. If meta-analyses are done, include for each, confidence intervals and measures of consistency.	8-12
Risk of bias across studies	22	Present results of any assessment of risk of bias across studies (see item 15).	-
Additional analyses	23	Give results of additional analyses, if done (such as sensitivity or subgroup analyses and metaregression [see item 16]).	8-12, 25-30
Discussion			
Summary of evidence	24	Summarize the main findings, including the strength of evidence for each main outcome; consider their relevance to key groups (such as healthcare providers, users, and policy makers).	12
Limitations	25	Discuss limitations at study and outcome level (such as risk of bias) and at review level (such as incomplete retrieval of identified research, reporting bias).	16
Conclusions	26	Provide a general interpretation of the results in the context of other evidence and implications for future research.	16
Funding			
Funding	27	Describe sources of funding or other support (such as supply of data) for the systematic review and the role of funders for the systematic review.	16

From Moher D, Liberati A, Tetzlaff J, and Altman DG; PRISMA group. Preferred Reporting Items for Systematic Reviews and Meta-analyses: the PRISMA statement. J Clin Epidemiol. 2009 Oct; 62 (10):1006-12. doi:10.1016/j.jclinepi.2009.06.005. Epub 2009 Jul 23. PMID: 19631508.

**Table 2:** Study characteristics of all articles included in meta-analysis.

Study year	Country	Mean age, Yrs (SD) KC vs. controls	Male (%) KC vs. controls	No. of eyes KC vs. controls	Representativeness of the case (%)	Design of studies	Materials	Test methods	Outcome	Contact lens worn (%) KC vs. controls
Lonescu 2018	Romania	23.35 (11.8) vs. 28.66 (3.03)	64.71% vs. 40%	17 vs. 15	Stage I (18%) Stage II (29%) Stage III (24%) Stage IV (29%)	Observational cross-sectional study	Tear fluids	LICA	IL-1*β*, INF-*γ*, IL-4, IL-6, IL-10, TNF-*α*	0
Pásztor 2016	American	44.2 vs. 44.5	NR	55 vs. 24	NR	Cross-sectional	Tear fluids	CBA	IL-6, IL-10, MMP-9, NGF	NR^∗^
Sorkhabi 2015	Iran	24.09 (6.50) vs. 24.43 (4.55)	57.14% vs. 43.33%	42 vs. 30	Mild (33.3%) Moderate (33.3%) Severe (33.3%)	Prospective case-controlled study	Tear fluids	ELISA	IL-1*β*, IL-6, IL-10	0
Balasubramanian 2012	Australia	27.4 (6.0) vs. 29.8 (8.9)	64% vs. 40%	25 vs. 20	NR	Cross-sectional study	Tear fluids	Cytokine antibody array	IL-1*β*, IL-4, IL-6, IL-10, TNF-*α*	NR^∗∗^
Lema 2009	Spain	27.1 (8.1) vs. 22.6 (6.6)	70% vs. 47.8%	30 vs. 20	NR	Case-control study	Tear fluids	ELISA	IL-6, TNF-*α*, MMP-9	56.7^∗∗∗^ vs. 0
Lema 2008	Spain	22.8 (6.6) vs. 22.6(6.6)	53.6% vs. 25%	28 vs. 20	NR	Case-control study	Tear fluids	ELISA	IL-6, IL-10, TNF-*α*	0
Lema 2005	Spain	22.4 (6.5) vs. 22.6 (6.6)	47.8% vs. 52.3%	28 vs. 20	Mild (21.4%) Moderate (50%) Severe (28.6%)	Case-control study	Tear fluids	ELISA	IL-4, IL-6, IL-10, TNF-*α*, MMP-9	0

**Table 3:** Methodological quality of case-control study using Newcastle-Ottawa Quality Assessment Scale.

Study year	Selection				Comparability	Exposure			Total
	Adequacy of case definition	Representativeness of case	Selection of controls	Definition of controls	Comparability of groups	Assessment of exposure	Methods of ascertainment/follow-up	Loss to follow-up/nonresponse rate	
Sorkhabi 2015	1	1	0	1	1	1	1	1	7
Lema 2009	1	0	0	1	1	1	1	1	6
Lema 2008	1	0	0	1	1	1	1	1	6
Lema 2005	1	1	0	1	1	1	1	1	7

For each item, star rating: a maximum of one star for each numbered item within the selection and exposure categories. A maximum of two stars can be given for comparability.

**Table 4:** Methodological quality of cross-sectional study using 11-item checklist of Agency for Healthcare Research and Quality.

Study year	Source of information	Inclusion and exclusion criteria of subjects	Time of identifying patients	Consecutiveness of subjects	Interference among outcomes	Assessment for quality assurance	Explain for exclusion	Assess and/or control confounding	Handle the miss data	Response rate of patients	Loss to follow-up	Total
Lonescu 2018	Y	Y	U	U	Y	U	Y	Y	Y	Y	Y	8
Pásztor 2016	Y	Y	Y	Y	Y	U	Y	Y	Y	Y	Y	10
Balasubramanian 2012	Y	Y	U	N	Y	U	Y	Y	Y	Y	Y	8

For each item, criteria fulfilled: N (no): 0; Y (yes): 1; U (unclear): 0.

**Table 5:** Results of metaregression analyses.

Cytokine	Heterogeneity factors	Coefficient	SE	*z*	*P*	95% CI (LCI, UCI)
IL-1*β*	Detection method	-2.78	0.471	-5.91	<0.001	(-3.702, -1.857)
	Unit of measurement	-1.406	2.377	-0.59	0.554	(-6.066, 3.253)
	Quality score	-2.78	0.471	-5.91	<0.001	(-3.702, -1.857)
	Region	-0.013	1.587	-0.01	0.993	(-3.123, 3.096)

IL-4	Detection method	3.573	3.939	0.91	0.363	(-4.128, 11.274)
	Unit of measurement	5.276	1.355	3.89	<0.001	(2.62, 7.932)
	Quality score	3.573	3.939	0.91	0.363	(-4.128, 11.274)
	Region	2.638	0.678	3.89	<0.001	(1.31, 3.966)

IL-6	Detection method	-0.784	0.509	-1.54	0.123	(-1.781, 0.214)
	Unit of measurement	0.212	0.874	0.24	0.808	(-1.5, 1.925)
	Quality score	0.177	0.681	0.26	0.795	(-1.158, 1.512)
	Region	0.506	0.259	1.95	0.051	(-0.003, 1.104)
	Age	-1.733	0.28	-6.19	<0.001	(-2.282, -1.184)

IL-10	Detection method	2.488	1.796	1.38	0.166	(-1.033, 6.008)
	Unit of measurement	3.976	1.718	2.31	0.021	(0.609, 7.343)
	Quality score	3.118	1.441	2.16	0.031	(0.292, 5.943)
	Region	0.797	1.012	0.79	0.431	(-1.187, 2.78)
	Age	-0.924	2.759	-0.33	0.738	(-6.331, 4.483)

TNF-*α*	Detection method	1.534	1.535	1.00	0.318	(-1.475, 4.543)
	Unit of measurement	3.661	0.898	4.08	<0.001	(1.901, 5.422)
	Quality score	1.534	1.535	1.00	0.318	(-1.475, 4.543)
	Region	1.831	0.449	4.08	<0.001	(0.95, 2.711)

SE: standard error; LCI: lower confidence interval; UCI: upper confidence interval.
